# Neuronal responses to cytokines limit intestinal hypermotility and systemic effects of colonic inflammation

**DOI:** 10.1016/j.isci.2026.116291

**Published:** 2026-06-08

**Authors:** Jisun Jung, Alec Wehmeier, Zili Xie, David Horton, Emilie V. Russler-Germain, Ellen Merrick Schill, Mark J. Miller, Jonathan R. Brestoff, Rodney Newberry, Brian S. Kim, Hongzhen Hu, Chyi-Song Hsieh

**Affiliations:** 1 Department of Internal Medicine, Division of Rheumatology, Washington University School of Medicine, St. Louis, MO, USA; 2 Department of Anesthesiology, The Center for the Study of Itch, Washington University School of Medicine, St. Louis, MO, USA; 3 Department of Pathology and Immunology, Washington University School of Medicine, St. Louis, MO, USA; 4 Department of Internal Medicine, Division of Gastroenterology, Washington University School of Medicine, St. Louis, MO, USA; 5 Division of Newborn Medicine, Department of Pediatrics, Washington University School of Medicine, St. Louis, MO, USA; 6 Department of Medicine, Washington University in St. Louis, St. Louis, MO, USA

**Keywords:** Molecular biology, Cellular neuroscience, Components of the immune system

## Abstract

Inflammation results in increased gastrointestinal (GI) motility and diarrhea, but the mechanism remains unclear. Contrary to expectations, we found that neuronal responses to cytokine inhibited GI motility, as Nav1.8-Cre-mediated neuronal deletion of Jak1, a cytokine signaling intermediate, markedly increased GI motility at homeostasis. These cytokine-responsive neurons may reside in the enteric nervous system (ENS) based on the pattern of gene-deletion and GI motility induced by Nav1.8- and Trpv1-Cre. Cytokine receptors upstream of Jak1 that inhibit GI motility at homeostasis include IFNγ and IL-10, but not IL-4 or IFNα. While deficiency in cytokine inhibition of GI motility did not appear to affect the health of the mice at homeostasis, worse outcomes were observed after experimental Dextran sulfate sodium (DSS) colitis. Thus, we speculate that IFNγ and IL-10, cytokines associated with type 1 immune responses, may participate in a protective neuro-immune negative feedback loop to limit excessive GI motility during intestinal inflammation.

## Highlights

• Nav1.8-cre expressing neurons limit gastrointestinal motility via Jak1 signaling
• Neuronal responses to Jak1-dependent cytokines IL-10 and IFNγ inhibit GI motility
• Neuronal responses to cytokines ameliorate DSS-induced colitis

## Graphical abstract

**Figure:**
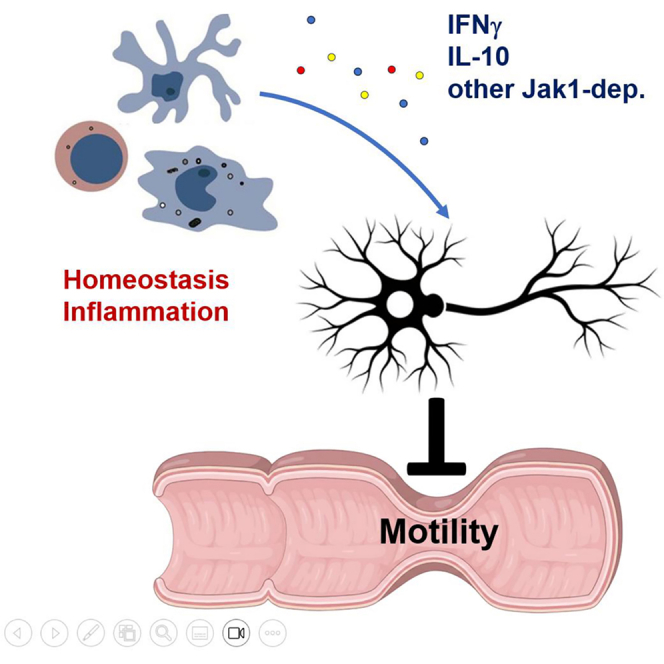

## Introduction

Gastrointestinal (GI) motility is the organized effort of coordinated smooth muscle moments^1^ which transits ingested food and fluids through the GI tract. Abnormal GI motility leading to diarrhea or constipation is a hallmark of irritable bowel syndrome (IBS),^2^ which affects approximately 6% of the US population with substantial effects on quality of life as well as economic impact.^3^^,^^4^

Consistent with reports suggesting an immune component to IBS,^5^^,^^6^ we previously found that human diarrhea-predominant IBS (IBS-D) is associated with altered adaptive IgA immune responses to gut bacteria compared with healthy controls.^7^ Analogous to the human data, we also found that an experimental murine stress model resulted in both diarrhea and changes to the IgA-bound bacteria. As prior studies have shown that the ratio of pro-vs. anti-inflammatory cytokines is increased in IBS-D,^8^^,^^9^ and that cytokines can affect GI motility,^10^^,^^11^^,^^12^ we hypothesized that stress-associated immune responses could induce GI motility changes via cytokines.

Cytokines could regulate GI motility indirectly via effects on mucosal barrier function.^13^ Alternatively, cytokines could directly affect neurons that regulate GI motility in a manner analogous to our previous observations in the skin, where cytokine signaling directly regulated itch via neurons in the dorsal root ganglion (DRG).^14^ Further support of this possibility came from a recent report that neurons in the DRG can also regulate intestinal immune responses to *Salmonella*.^15^ Although this study did not address GI motility, it supported the concept that immune-neuronal crosstalk can affect the GI tract.^16^^,^^17^

To test the hypothesis that cytokine signaling affects GI motility, we studied mice in which cytokine signaling mediators or receptors were selectively deleted in subsets of neurons in the peripheral and/or enteric nervous system (PNS/ENS). Contrary to our expectations, we found that GI motility is directly inhibited by neuronal responses to the Jak1-dependent cytokines IFNγ and IL10. Cytokine regulation of gut motility via neurons was observed during homeostasis and was associated with decreased weight loss in response to intestinal mucosal injury.

## Results

### Nav1.8-Cre expressing neurons regulate GI motility via Jak1-dependent signals

**Figure 1:**
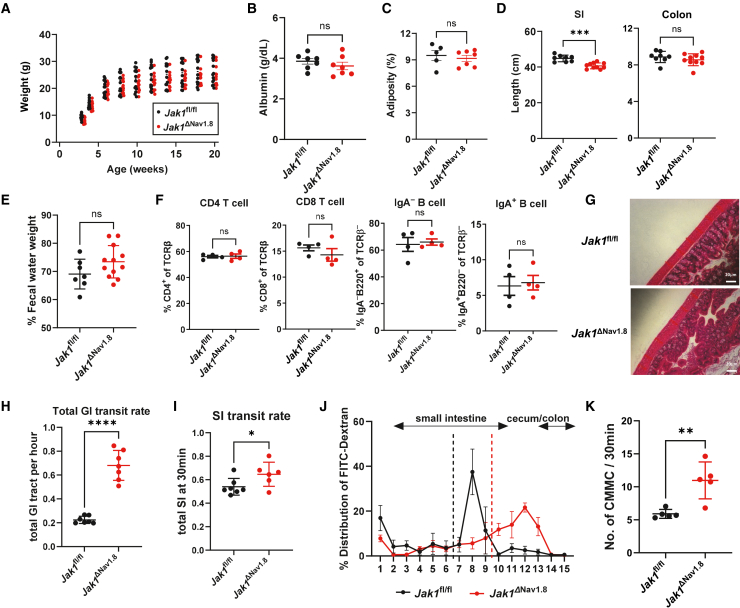
Responses to Jak1-mediated cytokine in Nav1.8-Cre^+^ neurons limits intestinal hypermotility Nav1.8-Cre^+^*Jak1*^fl/fl^ (*Jak1*^ΔNav1.8^) or littermate control *Jak1*^fl/fl^ mice were analyzed. (A) Body weight changes with age (*N* = 14 *Jak1*^fl/fl^, 12 *Jak1*^ΔNav1.8^, expt. = 2). (B) Serum albumin (*N* = 7 *Jak1*^fl/fl^, 7 *Jak1*^ΔNav1.8^, expt. = 2). (C) Adiposity (*N* = 5 *Jak1*^fl/fl^, 7 *Jak1*^ΔNav1.8^). (D) Length of the small intestine (left) or colon (right) (*N* = 8 *Jak1*^fl/fl^, 10 *Jak1*^ΔNav1.8^, expt. = 3). (E) Fecal water weight (*N* = 7 *Jak1*^fl/fl^, 12 *Jak1*^ΔNav1.8^, expt. = 3). (F) Immune cell phenotypes in the colon (*N* = 4 *Jak1*^fl/fl^, 4 *Jak1*^ΔNav1.8^, expt. = 2). (G) Representative histology of the proximal colon (*N* = 4 *Jak1*^fl/fl^, 4 *Jak1*^ΔNav1.8^). Scale bars represent 20 μm. (H) Total GI transit rate using carmine red dye gavage (*N* = 7 *Jak1*^fl/fl^, 7 *Jak1*^ΔNav1.8^, expt. = 2). (I) GI transit distance 30 min after carmine dye gavage, normalized to SI length (*N* = 7 *Jak1*^fl/fl^, 6 *Jak1*^ΔNav1.8^, expt. = 2). (J) Distribution of FITC-dextran along the GI tract 90 min after gavage. Fraction 1 assayed indicates stomach, 2–11 small intestine, 12 cecum and 13–15 colon (*N* = 5 *Jak1*^fl/fl^, 5 *Jak1*^ΔNav1.8^, expt. = 2). The vertical dash indicates the geometric center of the distribution of FITC-Dextran by (Σ[% FITC per segment × segment number])/100. (K) CMMC (*N* = 7 *Jak1*^fl/fl^, 6 *Jak1*^ΔNav1.8^). Mice were 7- to 10-week old unless otherwise noted. Each dot represents an individual mouse. *N* is the total number of mice. Significance was determined by two-way ANOVA (A) or unpaired two-tailed Student’s *t* test. Data are presented as mean ± SEM. ∗*p* < 0.05, ∗∗*p* < 0.01, ∗∗∗*p* < 0.001, ∗∗∗∗*p* < 0.0001. ns, not significant. See also Figure S1.

To test the hypothesis that Jak1-dependent cytokine signaling in neurons affects gut function, we studied Nav1.8-Cre *Jak1*^fl/fl^ (*Jak1*^ΔNav1.8^) mice.^14^^,^^18^ Consistent with prior reports, *Jak1*^ΔNav1.8^ mice appeared healthy compared to *Jak1*^fl/fl^ littermates, which was supported by assessments of weight gain over time, serum albumin, and measurements of adiposity (Figures 1A–1C). In terms of the intestines, we observed a slightly shorter small intestine (SI), but not colon, length in *Jak1*^ΔNav1.8^ mice compared with littermates (Figure 1D). However, the mice did not show signs of diarrhea by fecal water weight, differences in intestinal T cell/B cell subsets, or abnormality in histologic analysis (Figures 1E–1G).

Given these normal findings, we were surprised to observe a substantial increase in GI motility in *Jak1*^ΔNav1.8^ mice (Figure 1H), with total GI transit rate (1 GI tract length/total GI transit time) being approximately 3x faster than *Jak1*^fl/fl^ control mice. GI hypermotility was also seen in 3-week-old mice (Figure S1A). The feces from both ages of the mice showed non-significant changes in water weight between *Jak1*^fl/fl^ and *Jak1*^ΔNav1.8^ mice (Figure S1B). No sex differences were noted. Thus, these data reveal that *Jak1*^ΔNav1.8^ mice have GI hypermotility at homeostasis.

To assess whether this increased motility occurred in the SI, we determined the fraction of the SI the dye traveled 30 min after oral gavage.^9^ While we observed a statistically significant increase in this SI transit rate measurement in *Jak1*^ΔNav1.8^ mice (Figure 1I), this is a smaller change compared with the total GI transit rate (Figure 1H), suggesting that the reduction in GI transit time is greater in the distal small intestine and/or colon. We also quantified motility by examining the transit of FITC-dextran introduced by oral gavage.^19^^,^^20^ After 90 min, the GI tract was segmented and analyzed for fluorescence, which revealed that the peak of the dye was in the cecum/colon in *Jak1*^ΔNav1.8^ mice, but still within the SI in control mice (Figure 1J). To confirm changes in colonic motility, we assessed the colonic migrating motor complexes (CMMCs),^21^^,^^22^ which were substantially elevated in *Jak1*^ΔNav1.8^ mice (Figure 1K). Together, these data show that Jak1 in Nav1.8-Cre^+^ neurons provide an important inhibitory signal for intestinal motility predominantly in the colon.

### Nav1.8-Cre deletion occurs in multiple subsets of ENS neurons

**Figure 2:**
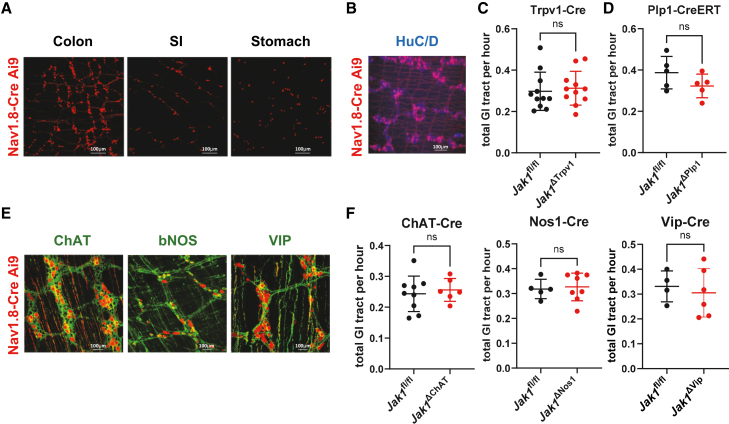
Nav1.8-Cre deletes in ENS neurons (A) TdTomato expression (red) in the GI tract of Nav1.8-Cre Ai9 mice using two photon microscopy. (*N* = 2, expt. = 2). (B) Colocalization of Nav1.8-Cre Ai9 tdTomato-expressing cells with HuC/D^+^ in the colonic myenteric plexus by immunofluorescence imaging. (*N* = 2, expt. = 2). (C) Total GI transit rate in *Jak1*^fl/fl^ crossed to Trpv1-Cre (*N* = 11 *Jak1*^fl/fl^, 11 *Jak1*^ΔTrpv1^, expt. = 3). (D) Total GI transit rate in *Jak1*^fl/fl^ crossed to Plp1-CreERT with daily i.p. injection of 100 mg/kg tamoxifen for 5 days (*N* = 5 *Jak1*^fl/fl^, 5 *Jak1*^ΔPlp1^, expt. = 2). (E) Colocalization of Nav1.8-Cre Ai9 tdTomato with ChAT (left), bNOS (middle), or VIP (right) in colonic myenteric plexus by immunofluorescence. (*N* = 2, expt. = 2). (F) Total GI transit rate in *Jak1*^fl/fl^ crossed to ChAT-Cre (*N* = 9 *Jak1*^fl/fl^, 6 *Jak1*^ΔChAT^, expt. = 2), Nos1-Cre (*N* = 5 *Jak1*^fl/fl^, 8 *Jak1*^ΔNos1^, expt. = 2), or Vip-Cre (*N* = 4 *Jak1*^fl/fl^, 6 *Jak1*^ΔVip^, expt. = 2) as indicated. Scale bars represent 100 μm (A, B, and E). Mice were 7- to 10-week old. Each dot represents an individual mouse. *N* is the total number of mice. Significance was determined by unpaired two-tailed Student’s *t* test. Data are presented as mean ± SEM. ns, not significant. See also Figure S2.

Nav1.8 is thought to be primarily expressed in DRG neurons, which may regulate gut motility via axonal projections to the gut.^15^ However, Nav1.8-Cre has also been reported to delete in enteric neurons.^23^ We therefore bred the Nav1.8-Cre mice with Ai9, confirming tdTomato (tdT) expression in the stomach, small intestine, and colon^23^ by fluorescent or two-photon microscopy (Figure 2A). Consistent with prior reports of *Scn10a* gene expression, we observed tdT^+^ fibers in the heart^24^^,^^25^^,^^26^^,^^27^ and scattered cells deep in the retina or optic nerve^28^ by two-photon microscopy (Figure S2A). Subjectively, more tdT expression was observed in the colon versus the SI or stomach, consistent with the increased density of the colonic myenteric plexus. The morphology and distribution of tdT suggested that they were neuron cell bodies and axons. Consistent with this, tdT^+^ cells co-stained with the neuronal marker HuC/D and were found within the colonic myenteric plexus (Figure 2B)^29^^,^^30^, although every locus differs in its deletion efficiency and does not always track with Ai9. Thus, the inhibitory effect of Jak1 on gut motility may be mediated via DRG and/or ENS neurons.

To distinguish between these possibilities, we used the Trpv1-Cre strain that has been reported to delete in DRG and not ENS neurons.^31^ Total GI transit rate was similar in *Jak1*^ΔTrpv1^ compared with *Jak1*^fl/fl^ littermates (Figure 2C), inferring that Jak1 expression on ENS neurons regulates GI motility.

As glial cells are abundant in the ENS,^32^ we also asked whether they may be involved in cytokine regulation of GI motility. However, the total GI transit rate was unchanged in tamoxifen treated Plp1-CreERT *Jak1*^fl/fl^ mice with conditional deletion of Jak1 in glial cells (Figure 2D). However, it remains possible that Plp1-Cre does not express in all glial cell subtypes and the number of enteric glia that would not be targeted by Plp1-CreERT is extremely low.^33^^,^^34^^,^^35^

We next asked if Nav1.8-Cre deleted in specific subsets of ENS neurons. We observed co-localization of tdT with neurons expressing choline acetyltransferase (ChAT), nitric oxide synthase (NOS), and to a lesser extent, vasoactive intestinal polypeptide (VIP) (Figure 2E).^29^^,^^30^^,^^36^^,^^37^ That Nav1.8-Cre mediated deletion occurs in multiple different ENS neuronal subtypes implies that Cre expression occurred early in the development of the ENS as most enteric neurons do not show gene expression of Nav1.8 in adults.^23^^,^^38^

We then asked if a specific neuronal subtype was responsible for Jak1-mediated inhibition of GI motility. However, Nos1-, Chat-, and Vip-Cre bred to *Jak1*^fl/fl^ mice (*Jak1*^ΔNos1^, *Jak1*^ΔChat^, *Jak1*^ΔVip^) did not show altered GI motility (Figure 2F). Together, these data suggest that Jak1 inhibits GI motility either via a subset of ENS neurons that does not express Nos, Chat, or VIP, or that these neuronal subsets are individually redundant for regulating GI motility.

### IFNγ and IL-10, but not IFNα or IL-4, inhibit GI motility at homeostasis

We next wished to define the cytokines that regulate GI motility via Jak1, a signaling kinase downstream of more than ten cytokines, including “γc” receptor cytokines, IL-6, and IFNγ.^16^^,^^39^^,^^40^ We focused on cytokines that have been reported to be expressed in ENS neurons^16^^,^^38^^,^^41^ or have been reported to affect GI motility via unclear mechanisms.^10^ These included the canonical Th1 and Th2 cytokines, IFNγ and IL-4, respectively, which have been reported to decrease and increase GI motility, respectively.^10^^,^^11^^,^^12^ We also examined IL-10, an important regulatory cytokine in the gut produced by Treg, Tr1, and myeloid cells, that has been reported to increase GI motility.^8^^,^^9^ Finally, we studied IFNα, a cytokine that is clearly induced in the intestine in response to microbiota^42^ and would likely be induced during viral gastroenteritis.^43^ *Jak1*, *Ifngr1*, *Il4ra*, and *Ifnar1* gene expression is detected in all colonic enteric neurons and glial subsets (Figure S2B) in a published scRNAseq dataset.^38^ *Il10ra* is not clearly detected but is poorly expressed even in immune cells known to respond to IL-10 (Figure S2B). In summary, we asked whether the Jak1-dependent cytokines IFNγ, IL-4, IL-10, or IFNα, whose receptors have been reported to be transcriptionally expressed in ENS neurons,^16^ modulate effects on GI motility via ENS neurons.

**Figure 3:**
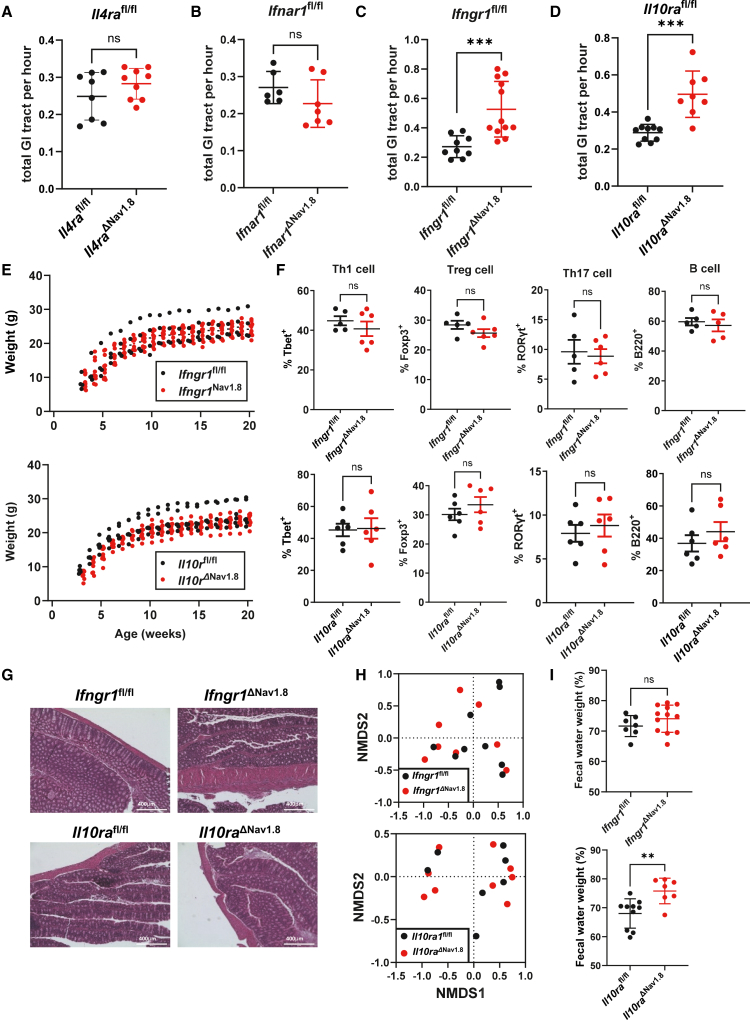
Responses to IFNγ and IL-10 by Nav1.8-Cre^+^ neurons limits GI motility (A–D) Total GI transit in Nav1.8-Cre mice crossed with (A) *Il4ra*^*fl/fl*^ (*N* = 8 *Il4ra*^*fl/fl*^, 9 *Il4ra*^ΔNav1.8^, expt. = 2); (B) *Ifnar1*^*fl/fl*^ (*N* = 6 *Ifnar1*^*fl/fl*^, 7 *Ifnar1*^ΔNav1.8^, expt. = 2); (C) *Ifngr1*^*fl/fl*^ (*N* = 9 *Ifngr1*^*fl/fl*^, 12 *Ifngr1*^ΔNav1.8^, expt. = 2); or (D) *Il10ra*^*fl/fl*^ (*N* = 10 *Il10ra*^*fl/fl*^, 8 *Il10ra*^ΔNav1.8^, expt. = 3). (E–I) Additional analyses of Nav1.8-Cre crossed to *Ifngr1*^*fl/fl*^ (top) or *Il10ra*^*fl/f*^ (bottom) mice. (E) Body weight (*N* = 4 *Ifngr1*^*fl/fl*^, 10 *Ifngr1*^ΔNav1.8^; 6 *Il10ra*^*fl/fl*^, 9 *Il10ra*^ΔNav1.8^, expt. = 2). (F) Immune cell phenotype in the colon (*N* = 5 *Ifngr1*^*fl/fl*^, 6 *Ifngr1*^ΔNav1.8^; 6 *Il10ra*^*fl/fl*^, 6 *Il10ra*^ΔNav1.8^, expt = 2). (G) Representative histology of the proximal colon (*N* = 4 each). Scale bars represent 400 μm. (H) Non-metric Multidimensional Scaling plots of Bray-Curtis dissimilarity of 16*S* rRNA sequencing of the terminal fecal pellet (*N* = 9 *Ifngr1*^*fl/fl*^, 9 *Ifngr1*^ΔNav1.8^; 7 *Il10ra*^*fl/fl*^, 9 *Il10ra*^ΔNav1.8^). (I) Fecal water weight (*N* = 7 *Ifngr1*^*fl/fl*^, 12 *Ifngr1*^ΔNav1.8^; 10 *Il10ra*^*fl/fl*^, 7 *Il10ra*^ΔNav1.8^, expt. = 2 each). Mice were 7- to 10-week old or as indicated. Each dot represents an individual mouse. *N* is the total number of mice. Significance was determined by unpaired two-tailed Student’s *t* test. Data are presented as mean ± SEM. ∗∗*p* < 0.01, ∗∗∗*p* < 0.001, ∗∗∗∗*p* < 0.0001. ns, not significant. See also Figures S3 and S4.

Nav1.8-Cre was bred to the respective floxed cytokine receptor mice and total GI transit rate at homeostasis was determined. *Il4ra*^ΔNav1.8^ mice showed normal GI transit (Figure 3A), which may be predicted as IL-4 is thought to be low at baseline in C57/BL6 background without external provocation.^44^^,^^45^^,^^46^ *Ifnar1*^ΔNav1.8^ mice also did not show altered GI transit (Figure 3B) despite the reports that IFNαR is expressed in ENS neurons and IFNα is induced in the gut by microbiota.^42^^,^^43^ However, these negative data suggest that GI hypermotility in *Jak1*^ΔNav1.8^ is not caused by Nav1.8-Cre neurotoxicity.

By contrast, *Ifngr1*^ΔNav1.8^ as well as *Il10ra*^ΔNav1.8^ mice showed increased GI motility (Figures 3C and 3D), phenocopying the *Jak1*^ΔNav1.8^ mice (Figure 1H). We attempted to address the cellular source of these cytokines for regulating GI motility. We corroborated the role of IFNγ in GI motility, observing that *Ifngr1*^−/−^ mice also showed increased transit rate (Figure S3A). However, we did note that *Ifngr1*^−/−^ mice weighed less than *Ifngr1*^+/−^ littermates (Figure S3B), suggesting unsurprisingly that IFNγ has additional functions in immune homeostasis beyond regulating GI motility. We therefore decided not to test *Il10*^−/−^ mice as they exhibit spontaneous colitis due to a loss of gut tolerance,^47^^,^^48^ which will likely have secondary effects on gut motility. Thus, defining the cellular source of cytokines that signal the ENS and regulate gut motility remains challenging.

Consistent with the *Jak1*^ΔNav1.8^ mice (Figure 1), we also did not observe baseline differences in weight in *Ifngr1*^ΔNav1.8^ or *Il10ra*^ΔNav1.8^ mice (Figure 3E). In addition, we did not observe marked evidence of inflammation based on flow cytometric analysis of T and B cell subsets or histology (Figures 3F and 3G; Figure S4). Furthermore, there were no marked differences in the microbiota based on 16*S* rRNA profiling (Figure 3H). Thus, these data suggest that IFNγ and IL-10 limit GI motility via Jak1-dependent signaling in neurons rather than via secondary effects on gut homeostasis.

One interesting observation was that *Il10ra*^ΔNav1.8^ mice showed increased fecal water weight, whereas *Ifngr1*^ΔNav1.8^, like *Jak1*^ΔNav1.8^, was unchanged (Figure 3I). This is reminiscent of the vastly divergent effects of IFNγ vs. IL-10 on immune cells (i.e., pro- vs. anti-inflammatory) even though both utilize Jak1 as an intermediate.^39^^,^^40^ Thus, these data imply that IL-10 and IFNγ have overlapping effects on ENS neurons to inhibit GI motility but may also exert distinct effects in terms of regulating water reabsorption or mucosal integrity.

### Neuronal cytokine responses ameliorate the effects of mucosal injury

**Figure 4:**
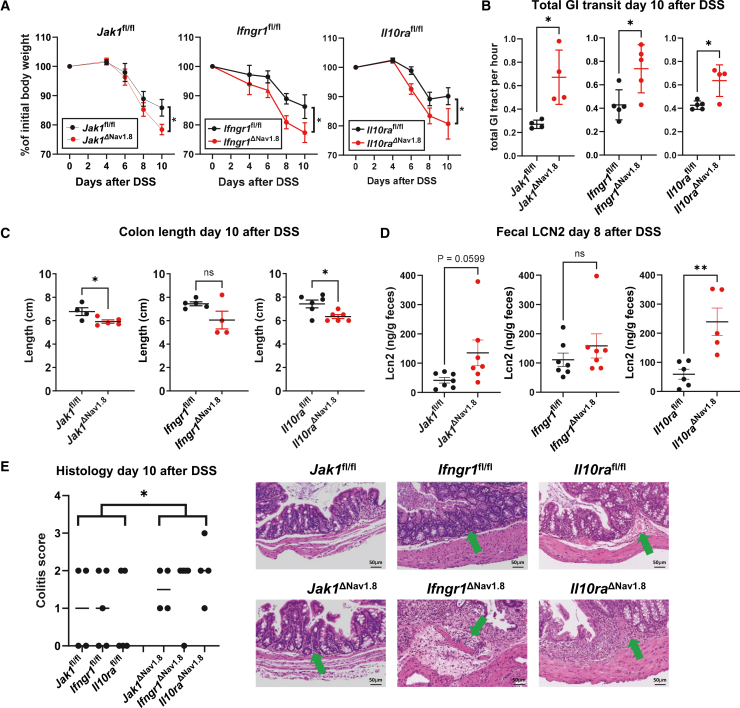
Responses to cytokines by Nav1.8-Cre expressing neurons limit the severity of intestinal injury Ten-week-old Nav1.8-Cre mice bred to *Jak1*^fl/fl^, *Ifngr1*^fl/fl^, or *Il10ra*^fl/fl^ were given 2% DSS in drinking water for 7 days. (A) Body weight as percentage of starting weight (*N* = 9 *Jak1*^fl/fl^, 12 *Jak1*^ΔNav1.8^, expt. = 3; 7 *Ifngr1*^*fl/fl*^, 7 *Ifngr1*^ΔNav1.8^, expt. = 2; 14 *Il10ra*^*fl/fl*^, 12 *Il10ra*^ΔNav1.8^, expt. = 3). (B) Total GI transit rate on day 10 after start of DSS (*N* = 4 *Jak1*^fl/fl^, 4 *Jak1*^ΔNav1.8^; 5 *Ifngr1*^*fl/fl*^, 5 *Ifngr1*^ΔNav1.8^; 5 *Il10ra*^*fl/fl*^, 4 *Il10ra*^ΔNav1.8^, expt. = 2). (C) Colon length on day 10 after start of DSS (*N* = 4 *Jak1*^fl/fl^, 5 *Jak1*^ΔNav1.8^; 5 *Ifngr1*^*fl/fl*^, 4 *Ifngr1*^ΔNav1.8^; 6 *Il10ra*^*fl/fl*^, 6 *Il10ra*^ΔNav1.8^). (D) Lcn2 of terminal fecal pellet on day 8 after start of DSS (*N* = 7 *Jak1*^fl/fl^, 7 *Jak1*^ΔNav1.8^; 7 *Ifngr1*^*fl/fl*^, 7 *Ifngr1*^ΔNav1.8^; 6 *Il10ra*^*fl/fl*^, 5 *Il10ra*^ΔNav1.8^, expt. = 2 each). (E) Histologic analysis of the proximal colon on day 10 after DSS. (Left) Summary of inflammation scores (range 0–3, see Method details; *N* = 4 *Jak1*^fl/fl^, 4 *Jak1*^ΔNav1.8^; 5 *Ifngr1*^*fl/fl*^, 5 *Ifngr1*^ΔNav1.8^; 5 *Il10ra*^*fl/fl*^, 4 *Il10ra*^ΔNav1.8^, expt. = 2). Statistics were performed on combined Nav1.8-Cre^–^ vs. Cre^+^ data. (Right) Representative histology of the tissues from colitis scores = 0 Jak1^fl/fl^, 1 *Jak1*^ΔNav1.8^; 1 *Ifngr1*^*fl/fl*^, 2 *Ifngr1*^ΔNav1.8^; 0 *Il10ra*^*fl/fl*^, 2 *Il10ra*^ΔNav1.8^. Arrows indicate areas of inflammation (score 1, neutrophils in lamina propria; score 2, plasma cells and lymphocytes in submucosa). Scale bars represent 50 μm. Each dot represents an individual mouse. *N* is the total number of mice. Significance was determined by two-way ANOVA (A) or unpaired two-tailed Student’s *t* test. Data are presented as mean ± SEM. ∗*p* < 0.05. ns, not significant. See also Figure S5.

Together, these data revealed that Nav1.8-Cre mediated conditional deletion of cytokine signaling by IFNγ or IL-10 results in a marked increase in GI motility at homeostasis without obvious inflammation, microbial changes, or impact on host health. We therefore asked whether the loss of cytokine signaling in Nav1.8-Cre expressing neurons would render the mice susceptible to DSS-induced mucosal injury in the colon. Notably, all three cytokine-signaling deficient strains of mice showed increased susceptibility to DSS with increased weight loss (Figure 4A). The increased total GI transit rate seen at homeostasis was maintained during colitis (Figures 4B and S5A) and could contribute to weight loss. Weight loss was also correlated with increased inflammation as evidenced by shorter colonic length, elevated fecal lipocalin 2 (Lcn2) levels, and inflammation by histology when the Nav1.8-Cre^+^ and ^–^ genotypes were pooled (Figures 4C–4E). However, flow cytometric profiling at day 10 did not reveal marked differences in T or B cell frequency or cell numbers between genotypes (Figure S5B). *Ifngr1*^ΔNav1.8^ mice showed a milder phenotype and did not reach statistical significance in some assays, reinforcing the notion that IL-10 and IFNγ have overlapping but also distinct effects on neurons impacted by Nav1.8-Cre. Thus, neuronal responses to cytokines may limit DSS colitis via regulation of intestinal inflammation^49^ as well as gut motility.

## Discussion

This study highlights the importance of neuronal cytokine responses for regulating GI motility and ameliorating the response to colonic inflammation. One limitation of these results is that it remains possible that neuronal responses to cytokine contribute toward other uncharacterized responses beyond peristalsis that indirectly affect GI motility or limits colonic inflammation and weight loss. It also remains possible that impaired responses to cytokine during neuronal development may mis-wire the circuits that regulate motility, although obvious neuronal alterations were not observed by light microscopy.

Another unresolved question from this study is the neuronal cell type(s) that sense Jak1-dependent cytokines and regulate GI motility. Our genetic experiments argue that these neurons reside in the ENS, but definitive proof would require a Cre-deleter strain specific to the ENS. We tested available Cre strains that delete subsets of ENS neurons, which excluded VIP, NOS, and Chat-expressing neurons as individually required for cytokine-mediated regulation of GI motility. However, this analysis is not comprehensive as a recent single cell RNAseq data of the ENS suggested that there are 12 distinct subsets in the small intestine.^38^

The cells affected by Nav1.8-Cre in the ENS remain an open question. While Ai9 expression was concordant with Nav1.8 expression in the tissues examined elsewhere in the body, this does not appear to be true in the intestine. One possible explanation is that as both ENS neurons and DRG neurons originate from neural crest progenitors, it is plausible that Nav1.8-Cre transiently label during development a subset of ENS neurons which downregulate expression of Scn10a later in life. This could also account for the absence of Nav1.8 detection in ENS RNA-seq datasets, while Nav1.8-Cre Ai9 mice still exhibit tdTomato expression in a subset of ENS neurons. Assumptions of Cre activity concordance with gene expression may affect the interpretation of prior studies of the intestine using this Cre-driver.^23^ Early deletion during development could result in a developmental mis-wiring that is not apparent by our imaging. Additionally, it remains possible that Nav1.8-Cre deletes in interstitial cells of Cajal, which are involved in GI motility but not known to express *Scn10a*.^50^ Determination of the cytokine-responsive neurons will be required to address the mechanisms by which cytokines affect motility such as Ca^2+^ flux^51^^,^^52^^,^^53^ and if differential regulation of cytokine receptor expression occurs during homeostasis and inflammation.^54^^,^^55^^,^^56^

Other cytokines may also contribute to neuronal regulation of GI motility. While we did not observe an effect of IL-4Rα deletion on GI motility at homeostasis, it is possible that Th2-responses during a worm infection or food allergy may trigger IL-4-dependent neuronal regulation of gut motility. We were also unable to define the source of the cytokines such as IFNγ or IL-10 that stimulate ENS neurons, as discriminating between cytokine effects on neurons vs. immune cells remains a challenge. Future studies are required to define the pathways of cytokine regulation of neuronal control of GI motility.

The recognition that Jak1-dependent cytokines can limit GI motility via neurons may be relevant to the use of Jak1 inhibitors clinically, as one of the major side effects includes diarrhea.^57^^,^^58^ However, diarrhea is not a universal phenomenon in patients, which may be due to the drug dose or to the combined effects of Jak1 inhibitors on the immune and nervous system. Future studies are required to determine whether Jak1 inhibitors can enhance GI motility via the nervous system in humans.

Although much remains to be discovered regarding the “circuitry” of Jak1-cytokine regulation of GI motility, one notable observation is that elimination of either responses to IFNγ or IL-10 is sufficient to result in hypermotility and increase severity of experimental colitis. This implies that neuronal inhibition of GI motility by these cytokines is controlled by “AND” logic. We speculate that this “AND” logic may favor inhibition of GI motility under conditions of high IFNγ and IL-10 that occurs during chronic Th1 inflammation,^59^ and reduction of water loss due to diarrhea during inflammation.

### Limitations of the study

While this study suggests that cytokine responses in neurons are important for regulating intestinal motility, the location and type of Nav1.8-Cre^+^ neurons important for cytokine-regulation of motility have not been definitively identified, which will require the use of new Cre drivers. Such information will be needed to determine the cellular source of cytokines in this pathway, as well as the relative importance of cytokine-mediated neuronal regulation of motility versus other neuronal and immunological responses during inflammation. Finally, deconvoluting the effects of cytokines on neurons vs. immune cells will be required to assess whether pharmacologic manipulation may be useful to treat human diseases associated with gut motility.

## Resource availability

### Lead contact

Requests for further information and resources should be directed to and will be fulfilled by the lead contact, Chyi-Song Hsieh (chsieh@wustl.edu).

### Materials availability

This study did not generate new unique reagents.

### Data and code availability

All data needed to evaluate the conclusions in the study are present in the paper or Supplementary materials. 16*S* sequencing data have been deposited at the European Nucleotide Archive (ENA) and are publicly available under accession number ENA: PRJEB112378.

## Acknowledgments

We would like to thank Nicole Santacruz and Patricia Hsieh for technical assistance. R.N. and C.-S.H. were funded by R01-AI140755.

## Author contributions

J.J., A.W., B.S.K., H.H., and C.-S.H. conceived the project. C.-S.H. supervised the study. J. J., A.W., and C.-S.H. designed experiments. J.J. and A.W. performed experiments and did data analysis. Z.X., D.H., E.V.R.-G., and E.M.S. provided technical support. M.J.M., J.R.B., and R.N. provided insightful discussions and supported experiments. J.J., A.W., and C.-S.H. wrote the manuscript. All authors reviewed and provided edits of the manuscript.

## Declaration of interests

The authors declare no competing interests.

## STAR★Methods

### Key resources table

**Table:** 

REAGENT or RESOURCE	SOURCE	IDENTIFIER
**Antibodies**		

anti-mouse HuC/D (16A11)	Invitrogen	Cat#A-21272
anti-sheep VIP	Millpore	Cat#AB1581
anti-sheep bNOS	Millpore	Cat#AB1529
anti-goat ChAT	Millpore	Cat#AB144P
anti-goat IgG Alexa 488	Invitrogen	A-11055
anti-sheep IgG FITC	Jackson	713-095-147
anti-mouse CD4 (RM4-5) APC	Biolegend	Cat#100516
anti-mouse TCRβ	Biolegend	Cat#140004
anti-mouse CD62L (MEL-14) PB	Biolegend	Cat#104424
anti-mouse/human CD44 (IM7) PECy7	Biolegend	Cat#103030
anti-mouse CD4 (RM4-5) PECy7	Biolegend	Cat#100528
anti-mouse CD8a (53-6.7) APCCy7	Biolegend	Cat#100714
anti-mouse FOXP3 (FJK-16s) FITC	ThermoFisher	Cat#11-5773-82
anti-mouse TBET (4B10) PECy7	Biolegend	Cat#644823
anti-mouse RORγt (B2D) PE	Invitrogen	Cat#12-6981-82
anti-B220	Biolegend	Cat#103232
anti-IgA	ThermoFisher	12-4204-82

**Chemicals, peptides, and recombinant proteins**		

FITC-Dextran	Millpore	Cat#46945
Dextran Sodium Sulfate	TdB Labs	Cat#9011-18-1

**Critical commercial assays**		

Propidium iodide	ThermoFisher	Cat#P3566
Quck-DNA fecal/soil Microbe Prep kit	Zymo	Cat#D6010
SuperScript III Reverse Transcriptase	ThermoFisher	Cat#18080093
Accuzyme DNA Polymerase	Bioline	Cat#BIO-21052
NucleoSpin RNA XS	Macherey-NAGEL	#740902.50
Foxp3/Transcription Factor Staining	ThermoFisher	Cat#00-5523-00

**Deposited data**		

16*S* rRNA	This paper	PRJEB112378

**Experimental models: Organisms/Strains**		

Nav1.8Cre	Rohini Kuner Lab	
Jak1 flox	Nanjing Biomedical Research Institute	
Ai9 (Rosa26-LSL-tdTomato)	Jackson Laboratory	
IL-4Rα flox	Frank Brombacher (ICGEB, Cape Town)	
IFNγR1 flox	Jackson Laboratory	
IFNαR1 flox	Deborah Lenschow Lab	
IL-10Rα flox	Jackson Laboratory	
Trpv1Cre	Jackson Laboratory	
Ifngr1^−/−^	Jackson Laboratory	
Plp1-CreERT	Jackson Laboratory	

**Software and algorithms**		

Prism v10	https://www.graphpad.com/	N/A
FlowJo v10	https://www.flowjo.com/	N/A
R v4.2	https://www.r-project.org/	N/A
data2 v3.15	https://www.bioconductor.org/packages/release/bioc/html/dada2.html	N/A
Phyloseq v3.18	https://www.bioconductor.org/packages/release/bioc/html/phyloseq.html	N/A

### Experimental model and study participant details

Mouse breeding and experiments were performed in an SPF facility using protocols approved by the Institutional Animal Care and Use Committees (IACUC) of Washington University in St. Louis (WashU). Nav1.8-Cre^14^^,^^18^ was bred with: *Jak1*^fl/fl^ (Model Animal Research Center of Nanjing University), *Ifngr1*^fl/fl^ (JAX:025394), *Ifnar1*^fl/fl^ (Deborah Lenschow at WashU),^60^ *Il4ra* ^fl/fl^ (Frank Brombacher at ICGEB, Cape Town),^61^ *Il10ra* ^fl/fl^ (JAX:028146), or Ai9 (JAX:007909). *Jak1*^fl/fl^ was also crossed to Nos1-Cre (JAX:017526), ChAT-Cre (JAX:031661), Vip-Cre (JAX:010908), Trpv1-Cre (JAX:003770), or Plp1-CreERT (JAX:005975). *Ifngr1*^−/−^ mice (JAX:003288) were mated with C57BL/6 (Charles River) and bred back to generate ^−/−^ and ^+/−^ littermates. New mouse stains transferred into the colony were transplanted one time with fecal microbiota from the Nav1.8-Cre *Jak1*^fl/fl^ line before breeding to Nav1.8-Cre *Jak1*^*fl/fl*^ mice to decrease effects of microbiota from various sources. Male and female littermates of Cre^+^ and Cre^–^ genotypes were used. Seven-to 10-week-old mice were used unless otherwise indicated.

#### Ethics statement

This study was carried out in strict accordance with the recommendations in the Guides for the Care and Use of Laboratory Animals of the National Institutes of Health (NIH). The protocols were approved by the Institutional Animal Care and Use Committee (IACUC) at Washington University School of Medicine (protocol ID: 25–0350).

### Method detailsdetails

#### GI transit measurements

Mice were single housed in clean mouse cages without bedding, gavaged with 200 mL of red transit dye (2.5% Methylcellulose (w/v) and 3% Carmine powder dye (Sigma Aldrich, c1022) in water (w/v), autoclaved after mixing. 100 mL was given to 3-week-old mice. The mice were monitored for time to expulsion of red stained feces (total GI transit time) or were sacrificed in 30 min after the gavage for SI transit distance. Total GI transit rate is defined as 1 GI tract/total GI transit time in hours. The SI transit rate is calculated from the fraction of the SI transited in 30 min. All the mice were given access to unlimited food and water during the experiment.

#### FITC-dextran assay

Mice were gavaged with 100 mL 70 kDa FITC-dextran (5 mg/mL, Sigma Aldrich, 46945) and sacrificed at 90 min. The small intestine or colon were cut into 10 or 3 equal segments, respectively. The contents of the stomach (#1) or intestinal segments: small intestine (#2–11), cecum (#12) or colon (#13–15), were flushed with 1 mL HBSS and the supernatants were collected. FITC intensity was determined by spectrophotometer at 494 nm/521 nm.

#### Colon migrating motor complexes

Colon contraction motility was measured as described^62^; Briefly, colons after removing fecal contents were placed in an organ bath chamber with physiological saline and attached to submerged flow tubes at each end. The chamber was maintained at 35 °C and aerated with carbogen. A video camera recorded the frequency of the colon contraction for 30 min.

#### Fecal water weight

The terminal fecal pellets of mice were collected in pre-weighed screw tubes (Tube_pre_). The tubes with fecal pellets and water were weighed (Tube_feces_) and placed open in an oven and dried overnight. The dry tubes with dried fecal pellets were weighed (Tube_dried_), emptied, and then the empty dry tubes were weighed (Tube_post_). Percent fecal water weight (FWW) is defined as $FWW=100\%\left(\frac{\left(\left({Tube}_{feces}-{Tube}_{pre}\right)-\left({Tube}_{dried}-{Tube}_{post}\right)\right)}{\left({Tube}_{feces}-{Tube}_{pre}\right)}\right)$

#### Antibodies and flow cytometry

Single cell suspensions from colonic lamina propria or mesenteric lymph nodes were isolated and washed with wash buffer (DMEM with 1% FBS). The cells were stained with the following antibody mixtures in FACS buffer (PBS with 2.5% FBS, 0.05% NaN_3_) for 20 min on ice. Anti-CD4 (RM4-5), anti-TCRβ, anti-CD62L (MEL-14), anti-CD44 (IM7), anti-CD8α (53-6.7) from BioLegend or anti-B220 (RA3-6B2), anti-IgA (mA-6E1) from Invitrogen. The cells were also permeabilized/fixed with eBioscience™ Foxp3/Transcription Factor Staining Buffer Set and stained with anti-Tbet (4B10) from BioLegend or anti-Foxp3 (FJK-16s), anti-RORγt (B2D) from Invitrogen. Data were collected using a FACSAria Ilu or Attune NxT Flow Cytometer.

#### Lamina propria lymphocyte isolation

Lymphocytes from the colon were isolated as previously described^63^; Briefly, the colon was harvested and cut into small pieces for incubation for 20 min at 37°C in PBS with 3% FBS, EDTA (10 mM), HEPES (20 mM), penicillin (100U/mL), streptomycin (100 mg/mL) and sodium pyruvate (1 mM) to remove epithelia cells. The tissue fragments were digested with collagenase D and DNase I in RPMI with 3% FBS, HEPES (20 mM), penicillin (100U/mL), streptomycin (100 mg/mL), sodium pyruvate (1 mM) and non-essential amino acids (1 mM). Lymphocytes were enriched via centrifugation at 1300*g* in 40:75% Percoll for 20 min at 25°C without brake.

#### Histology and colitis scoring

Proximal colon was collected and cut open longitudinally after removing luminal contents. The tissues were immediately fixed in 10% (vol/vol) formalin and then paraffin-embedded. Cut sections were stained with Hematoxylin and Eosin (H&E) (HCS Inc., Washington).

Inflammation in the colon was scored by a blinded pathologist (E.V.R.): 0, rare inflammatory cells in the lamina propria; 1, increased numbers of granulocytes in the lamina propria; 2, inflammatory cell aggregates in submucosa–serosal fat may have scattered inflammation; 3, robust transmural inflammation and captured transmural extension or dense serosal inflammation.

#### Whole mount fluorescent immunohistochemistry

Colons were pinned flat on sylgard plates and fixed with 4% paraformaldehyde. Myenteric and submucosal layers were separated by mechanical dissection. The myenteric layer was stained with purified anti-HuC/D (16A11), anti-sheep VIP, anti-sheep bNOS, or anti-goat ChAT, and then secondary anti-goat IgG Alexa 488 or anti-sheep FITC antibodies.

#### Tamoxifen treatment

Plp1-CreERT *Jak1*^*fl/fl*^ mice were injected i.p. with tamoxifen (100 μg/kg) in corn oil for 5 days daily, followed by a transit experiment 7 days after the last injection.

#### Acute DSS mouse model

For days 0–7 of the experiment, drinking water was replaced by 2% DSS in water (TdB Labs, DB001). Mice were monitored for survival every day and their body weights were recorded every other day until day 10. Mice were sacrificed if their body weights dropped below 80% of their original day 0 body weights.

#### 16*S* rRNA sequencing

16*S* sequencing was conducted as previously described.^63^ Fecal DNA was extracted using quick-DNA fecal microbe miniprep kit and the PCR product of the bacterial V4 region^64^ of 16*S* rRNA were sequenced using Illumina MiSeq (2x250-bp paired-end reads). ASVs and taxonomy including species designations if possible (silva 1.38) were determined by dada2.^65^

#### scRNA sequencing data acquisition

scRNA sequencing data were obtained from Single cell portal accession SCP1038 (https://singlecell.broadinstitute.org/single_cell/study/SCP1038/the-human-and-mouse-enteric-nervous-system-at-single-cell-resolution). Splenocyte data were from Single cell portal accession SCP306.

### Quantification and statistical analysis

Statistical analyses were performed using GraphPad Prism version 10. Data are presented as Mean and SEM unless otherwise indicated. Significance was typically determined by unpaired two-tailed Student’s *t* test**.** Repeated measures ANOVA was used for body weight loss analyses**.** Each dot represents data from an individual mouse. Statistical tests used for each experiment are indicated in figure legends. Statistical significance was defined by ∗*p* < 0.05, ∗∗*p* < 0.01, ∗∗∗*p* < 0.001, ∗∗∗∗*p* < 0.0001.

## References

[1] Sanders K.M., Koh S.D., Ro S., Ward S.M. (2012). Regulation of gastrointestinal motility--insights from smooth muscle biology. Nat. Rev. Gastroenterol. Hepatol..

[2] Almario C.V., Sharabi E., Chey W.D., Lauzon M., Higgins C.S., Spiegel B.M.R. (2023). Prevalence and Burden of Illness of Rome IV Irritable Bowel Syndrome in the United States: Results From a Nationwide Cross-Sectional Study. Gastroenterology.

[3] Kopczyńska M., Mokros Ł., Pietras T., Małecka-Panas E. (2018). Quality of life and depression in patients with irritable bowel syndrome. Prz Gastroenterol.

[4] Trindade I.A., Melchior C., Törnblom H., Simrén M. (2022). Quality of life in irritable bowel syndrome: Exploring mediating factors through structural equation modelling. J. Psychosom. Res..

[5] Guilarte M., Santos J., de Torres I., Alonso C., Vicario M., Ramos L., Martinez C., Casellas F., Saperas E., Malagelada J.R. (2007). Diarrhoea-predominant IBS patients show mast cell activation and hyperplasia in the jejunum. Gut.

[6] Dong Y., Han Y., Wang Z., Qin Z., Yang C., Cao J., Chen Y. (2017). Role of serotonin on the intestinal mucosal immune response to stress-induced diarrhea in weaning mice. BMC Gastroenterol..

[7] Rengarajan S., Knoop K.A., Rengarajan A., Chai J.N., Grajales-Reyes J.G., Samineni V.K., Russler-Germain E.V., Ranganathan P., Fasano A., Sayuk G.S. (2020). A Potential Role for Stress-Induced Microbial Alterations in IgA-Associated Irritable Bowel Syndrome with Diarrhea. Cell Rep. Med..

[8] Schmulson M., Pulido-London D., Rodriguez O., Morales-Rochlin N., Martinez-García R., Gutierrez-Ruiz M.C., López-Alvarenga J.C., Robles-Díaz G., Gutiérrez-Reyes G. (2012). Lower serum IL-10 is an independent predictor of IBS among volunteers in Mexico. Am. J. Gastroenterol..

[9] Liebregts T., Adam B., Bredack C., Röth A., Heinzel S., Lester S., Downie–Doyle S., Smith E., Drew P., Talley N.J., Holtmann G. (2007). Immune activation in patients with irritable bowel syndrome. Gastroenterology.

[10] Akiho H., Ihara E., Motomura Y., Nakamura K. (2011). Cytokine-induced alterations of gastrointestinal motility in gastrointestinal disorders. World J. Gastrointest. Pathophysiol..

[11] Akiho H., Lovato P., Deng Y., Ceponis P.J.M., Blennerhassett P., Collins S.M. (2005). Interleukin-4- and -13-induced hypercontractility of human intestinal muscle cells-implication for motility changes in Crohn's disease. Am. J. Physiol. Gastrointest. Liver Physiol..

[12] Ford C.L., Wang Y., Morgan K., Boktor M., Jordan P., Castor T.P., Alexander J.S. (2019). Interferon-gamma depresses human intestinal smooth muscle cell contractility: Relevance to inflammatory gut motility disturbances. Life Sci..

[13] Ahrends T., Aydin B., Matheis F., Classon C.H., Marchildon F., Furtado G.C., Lira S.A., Mucida D. (2021). Enteric pathogens induce tissue tolerance and prevent neuronal loss from subsequent infections. Cell.

[14] Oetjen L.K., Mack M.R., Feng J., Whelan T.M., Niu H., Guo C.J., Chen S., Trier A.M., Xu A.Z., Tripathi S.V. (2017). Sensory Neurons Co-opt Classical Immune Signaling Pathways to Mediate Chronic Itch. Cell.

[15] Lai N.Y., Musser M.A., Pinho-Ribeiro F.A., Baral P., Jacobson A., Ma P., Potts D.E., Chen Z., Paik D., Soualhi S. (2020). Gut-Innervating Nociceptor Neurons Regulate Peyer's Patch Microfold Cells and SFB Levels to Mediate Salmonella Host Defense. Cell.

[16] Jacobson A., Yang D., Vella M., Chiu I.M. (2021). The intestinal neuro-immune axis: crosstalk between neurons, immune cells, and microbes. Mucosal Immunol..

[17] Veiga-Fernandes H., Mucida D. (2016). Neuro-Immune Interactions at Barrier Surfaces. Cell.

[18] Agarwal N., Offermanns S., Kuner R. (2004). Conditional gene deletion in primary nociceptive neurons of trigeminal ganglia and dorsal root ganglia. Genesis.

[19] Woting A., Blaut M. (2018). Small Intestinal Permeability and Gut-Transit Time Determined with Low and High Molecular Weight Fluorescein Isothiocyanate-Dextrans in C3H Mice. Nutrients.

[20] Soni K.G., Halder T., Conner M.E., Preidis G.A. (2019). Sexual dimorphism in upper gastrointestinal motility is dependent on duration of fast, time of day, age, and strain of mice. Neuro Gastroenterol. Motil..

[21] Barnes K.J., Beckett E.A., Brookes S.J., Sia T.C., Spencer N.J. (2014). Control of intrinsic pacemaker frequency and velocity of colonic migrating motor complexes in mouse. Front. Neurosci..

[22] Smith T.K., Park K.J., Hennig G.W. (2014). Colonic migrating motor complexes, high amplitude propagating contractions, neural reflexes and the importance of neuronal and mucosal serotonin. J. Neurogastroenterol. Motil..

[23] Servin-Vences M.R., Lam R.M., Koolen A., Wang Y., Saade D.N., Loud M., Kacmaz H., Frausto S., Zhang Y., Beyder A. (2023). PIEZO2 in somatosensory neurons controls gastrointestinal transit. Cell.

[24] Han C., Huang J., Waxman S.G. (2016). Sodium channel Nav1.8: Emerging links to human disease. Neurology.

[25] Qi B., Wei Y., Chen S., Zhou G., Li H., Xu J., Ding Y., Lu X., Zhao L., Zhang F. (2014). Nav1.8 channels in ganglionated plexi modulate atrial fibrillation inducibility. Cardiovasc. Res..

[26] Chen X., Yu L., Shi S., Jiang H., Huang C., Desai M., Li Y., Barajas-Martinez H., Hu D. (2016). Neuronal Nav1.8 Channels as a Novel Therapeutic Target of Acute Atrial Fibrillation Prevention. J. Am. Heart Assoc..

[27] Gautron L., Sakata I., Udit S., Zigman J.M., Wood J.N., Elmquist J.K. (2011). Genetic tracing of Nav1.8-expressing vagal afferents in the mouse. J. Comp. Neurol..

[28] O'Brien B.J., Caldwell J.H., Ehring G.R., Bumsted O'Brien K.M., Luo S., Levinson S.R. (2008). Tetrodotoxin-resistant voltage-gated sodium channels Na(v)1.8 and Na(v)1.9 are expressed in the retina. J. Comp. Neurol..

[29] Parathan P., Wang Y., Leembruggen A.J., Bornstein J.C., Foong J.P. (2020). The enteric nervous system undergoes significant chemical and synaptic maturation during adolescence in mice. Dev. Biol..

[30] Li Z., Hao M.M., Van den Haute C., Baekelandt V., Boesmans W., Vanden Berghe P. (2019). Regional complexity in enteric neuron wiring reflects diversity of motility patterns in the mouse large intestine. eLife.

[31] Masuoka T., Kudo M., Yamashita Y., Yoshida J., Imaizumi N., Muramatsu I., Nishio M., Ishibashi T. (2017). TRPA1 Channels Modify TRPV1-Mediated Current Responses in Dorsal Root Ganglion Neurons. Front. Physiol..

[32] Progatzky F., Pachnis V. (2022). The role of enteric glia in intestinal immunity. Curr. Opin. Immunol..

[33] Baghdadi M.B., Ayyaz A., Coquenlorge S., Chu B., Kumar S., Streutker C., Wrana J.L., Kim T.H. (2022). Enteric glial cell heterogeneity regulates intestinal stem cell niches. Cell Stem Cell.

[34] Sanchini G., Vaes N., Boesmans W. (2023). Mini-review: Enteric glial cell heterogeneity: Is it all about the niche?. Neurosci. Lett..

[35] Rao M., Nelms B.D., Dong L., Salinas-Rios V., Rutlin M., Gershon M.D., Corfas G. (2015). Enteric glia express proteolipid protein 1 and are a transcriptionally unique population of glia in the mammalian nervous system. Glia.

[36] Lei C., Sun R., Xu G., Tan Y., Feng W., McClain C.J., Deng Z. (2022). Enteric VIP-producing neurons maintain gut microbiota homeostasis through regulating epithelium fucosylation. Cell Host Microbe.

[37] Beck M., Schlabrakowski A., Schrödl F., Neuhuber W., Brehmer A. (2009). ChAT and NOS in human myenteric neurons: co-existence and co-absence. Cell Tissue Res..

[38] Drokhlyansky E., Smillie C.S., Van Wittenberghe N., Ericsson M., Griffin G.K., Eraslan G., Dionne D., Cuoco M.S., Goder-Reiser M.N., Sharova T. (2020). The Human and Mouse Enteric Nervous System at Single-Cell Resolution. Cell.

[39] Hu X., Li J., Fu M., Zhao X., Wang W. (2021). The JAK/STAT signaling pathway: from bench to clinic. Signal Transduct. Target. Ther..

[40] Morris R., Kershaw N.J., Babon J.J. (2018). The molecular details of cytokine signaling via the JAK/STAT pathway. Protein Sci..

[41] Gougeon P.Y., Lourenssen S., Han T.Y., Nair D.G., Ropeleski M.J., Blennerhassett M.G. (2013). The pro-inflammatory cytokines IL-1beta and TNFalpha are neurotrophic for enteric neurons. J. Neurosci..

[42] Wirusanti N.I., Baldridge M.T., Harris V.C. (2022). Microbiota regulation of viral infections through interferon signaling. Trends Microbiol..

[43] Lam K.C., Araya R.E., Huang A., Chen Q., Di Modica M., Rodrigues R.R., Lopès A., Johnson S.B., Schwarz B., Bohrnsen E. (2021). Microbiota triggers STING-type I IFN-dependent monocyte reprogramming of the tumor microenvironment. Cell.

[44] Lee Y.J., Holzapfel K.L., Zhu J., Jameson S.C., Hogquist K.A. (2013). Steady-state production of IL-4 modulates immunity in mouse strains and is determined by lineage diversity of iNKT cells. Nat. Immunol..

[45] Nishimura T., Santa K., Yahata T., Sato N., Ohta A., Ohmi Y., Sato T., Hozumi K., Habu S. (1997). Involvement of IL-4-producing Vbeta8.2+ CD4+ CD62L- CD45RB- T cells in non-MHC gene-controlled predisposition toward skewing into T helper type-2 immunity in BALB/c mice. J. Immunol..

[46] Gregory G.D., Raju S.S., Winandy S., Brown M.A. (2006). Mast cell IL-4 expression is regulated by Ikaros and influences encephalitogenic Th1 responses in EAE. J. Clin. Investig..

[47] Kühn R., Löhler J., Rennick D., Rajewsky K., Müller W. (1993). Interleukin-10-deficient mice develop chronic enterocolitis. Cell.

[48] Iyer S.S., Cheng G. (2012). Role of interleukin 10 transcriptional regulation in inflammation and autoimmune disease. Crit. Rev. Immunol..

[49] da Silva Watanabe P., Cavichioli A.M., D'Arc de Lima Mendes J., Aktar R., Peiris M., Blackshaw L.A., de Almeida Araújo E.J. (2023). Colonic motility adjustments in acute and chronic DSS-induced colitis. Life Sci..

[50] Foong D., Zhou J., Zarrouk A., Ho V., O’Connor M.D. (2020). Understanding the Biology of Human Interstitial Cells of Cajal in Gastrointestinal Motility. Int. J. Mol. Sci..

[51] Sanders K.M. (2001). Invited review: mechanisms of calcium handling in smooth muscles. J. Appl. Physiol..

[52] Sanders K.M., Drumm B.T., Cobine C.A., Baker S.A. (2024). Ca(2+) dynamics in interstitial cells: foundational mechanisms for the motor patterns in the gastrointestinal tract. Physiol. Rev..

[53] Himpens B., Casteels R. (1990). Different effects of depolarization and muscarinic stimulation on the Ca2+/force relationship during the contraction-relaxation cycle in the guinea pig ileum. Pflugers Arch.

[54] Wang H., Foong J.P.P., Harris N.L., Bornstein J.C. (2022). Enteric neuroimmune interactions coordinate intestinal responses in health and disease. Mucosal Immunol..

[55] Magalhães H.I.R., Castelucci P. (2021). Enteric nervous system and inflammatory bowel diseases: Correlated impacts and therapeutic approaches through the P2X7 receptor. World J. Gastroenterol..

[56] Lakhan S.E., Kirchgessner A. (2010). Neuroinflammation in inflammatory bowel disease. J. Neuroinflammation.

[57] Honap S., Irving P.M., Samaan M.A. (2023). JAK inhibitors for the treatment of inflammatory bowel disease: results of an international survey of perceptions, attitudes, and clinical practice. Eur. J. Gastroenterol. Hepatol..

[58] Aguilar D., Revilla L., Garrido-Trigo A., Panés J., Lozano J.J., Planell N., Esteller M., Lacerda A.P., Guay H., Butler J. (2021). Randomized Controlled Trial Substudy of Cell-specific Mechanisms of Janus Kinase 1 Inhibition With Upadacitinib in the Crohn's Disease Intestinal Mucosa: Analysis From the CELEST Study. Inflamm. Bowel Dis..

[59] Buzoni-Gatel D., Werts C. (2006). Toxoplasma gondii and subversion of the immune system. Trends Parasitol..

[60] Cook L.E., Locke M.C., Young A.R., Monte K., Hedberg M.L., Shimak R.M., Sheehan K.C.F., Veis D.J., Diamond M.S., Lenschow D.J. (2019). Distinct Roles of Interferon Alpha and Beta in Controlling Chikungunya Virus Replication and Modulating Neutrophil-Mediated Inflammation. J. Virol..

[61] Dewals B., Hoving J.C., Leeto M., Marillier R.G., Govender U., Cutler A.J., Horsnell W.G.C., Brombacher F. (2009). IL-4Ralpha responsiveness of non-CD4 T cells contributes to resistance in schistosoma mansoni infection in pan-T cell-specific IL-4Ralpha-deficient mice. Am. J. Pathol..

[62] Swaminathan M., Hill-Yardin E., Ellis M., Zygorodimos M., Johnston L.A., Gwynne R.M., Bornstein J.C. (2016). Video Imaging and Spatiotemporal Maps to Analyze Gastrointestinal Motility in Mice. J. Vis. Exp..

[63] Russler-Germain E.V., Jung J., Miller A.T., Young S., Yi J., Wehmeier A., Fox L.E., Monte K.J., Chai J.N., Kulkarni D.H. (2021). Commensal Cryptosporidium colonization elicits a cDC1-dependent Th1 response that promotes intestinal homeostasis and limits other infections. Immunity.

[64] Caporaso J.G., Lauber C.L., Walters W.A., Berg-Lyons D., Lozupone C.A., Turnbaugh P.J., Fierer N., Knight R. (2011). Global patterns of 16S rRNA diversity at a depth of millions of sequences per sample. Proc. Natl. Acad. Sci. USA.

[65] Callahan B.J., McMurdie P.J., Rosen M.J., Han A.W., Johnson A.J.A., Holmes S.P. (2016). DADA2: High-resolution sample inference from Illumina amplicon data. Nat. Methods.

