# Supplementary material for: Neuronal responses to cytokines limit intestinal hypermotility and systemic effects of colonic inflammation
Source: iScience. 2026 Jun 8;29(6):116291. doi: 10.1016/j.isci.2026.116291 (PMC13264127; doi:10.1016/j.isci.2026.116291)
Supplement: Document S1. Figures S1–S5 [file mmc1.pdf]

iScience, Volume *29*

Supplemental information

Neuronal responses to cytokines limit intestinal hypermotility and systemic effects of colonic inflammation

Jisun Jung, Alec Wehmeier, Zili Xie, David Horton, Emilie V. Russler-Germain, Ellen Merrick Schill, Mark J. Miller, Jonathan R. Brestoff, Rodney Newberry, Brian S. Kim, Hongzhen Hu, and Chyi-Song Hsieh

Supplemental information

Neuronal responses to cytokine limit intestinal hypermotility and systemic effects of colonic inflammation

Jisun Jung, Alec Wehmeier, Zili Xie, David Horton, Emilie V Russler-Germain, Ellen Merrick Schill, Mark J. Miller, Jonathan R Brestoff, Rodney Newberry, Brian S. Kim,* Hongzhen Hu,* and Chyi-Song Hsieh*

***Corresponding:** chsieh@wustl.edu, hongzhen.hu@mssm.edu, itchdoctor@mountsinai.org

The PDF file includes:

Figure S1 to S5

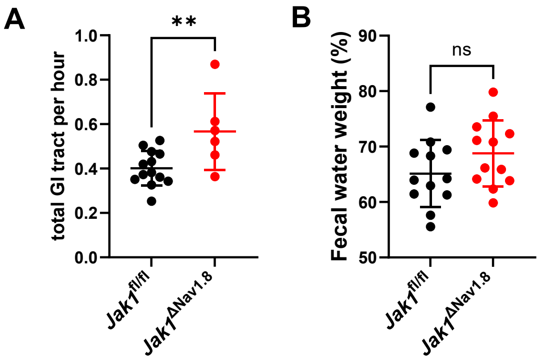

Figure S1. Rapid GI transit in 3-week-old Nav1.8-Cre *Jak1*$^{fl/fl}$ mice, related to Figure 1

Three-week-old *Jak1*$^{fl/fl}$ or *Jak1*$^{\Delta Nav1.8}$ mice were analyzed.

(A) Total GI transit (*N*=13 *Jak1*$^{fl/fl}$, 6 *Jak1*$^{\Delta Nav1.8}$, expt.= 2).

(B) Fecal water weight (*N*=11 *Jak1*$^{fl/fl}$, 13 *Jak1*$^{\Delta Nav1.8}$, expt.= 2).

Each dot represents an individual mouse. *N* is the total number of mice.

Significance was determined by unpaired two-tailed Student's *t* test. Data are presented as mean ±SEM.

**P*<0.05. ns, not significant.

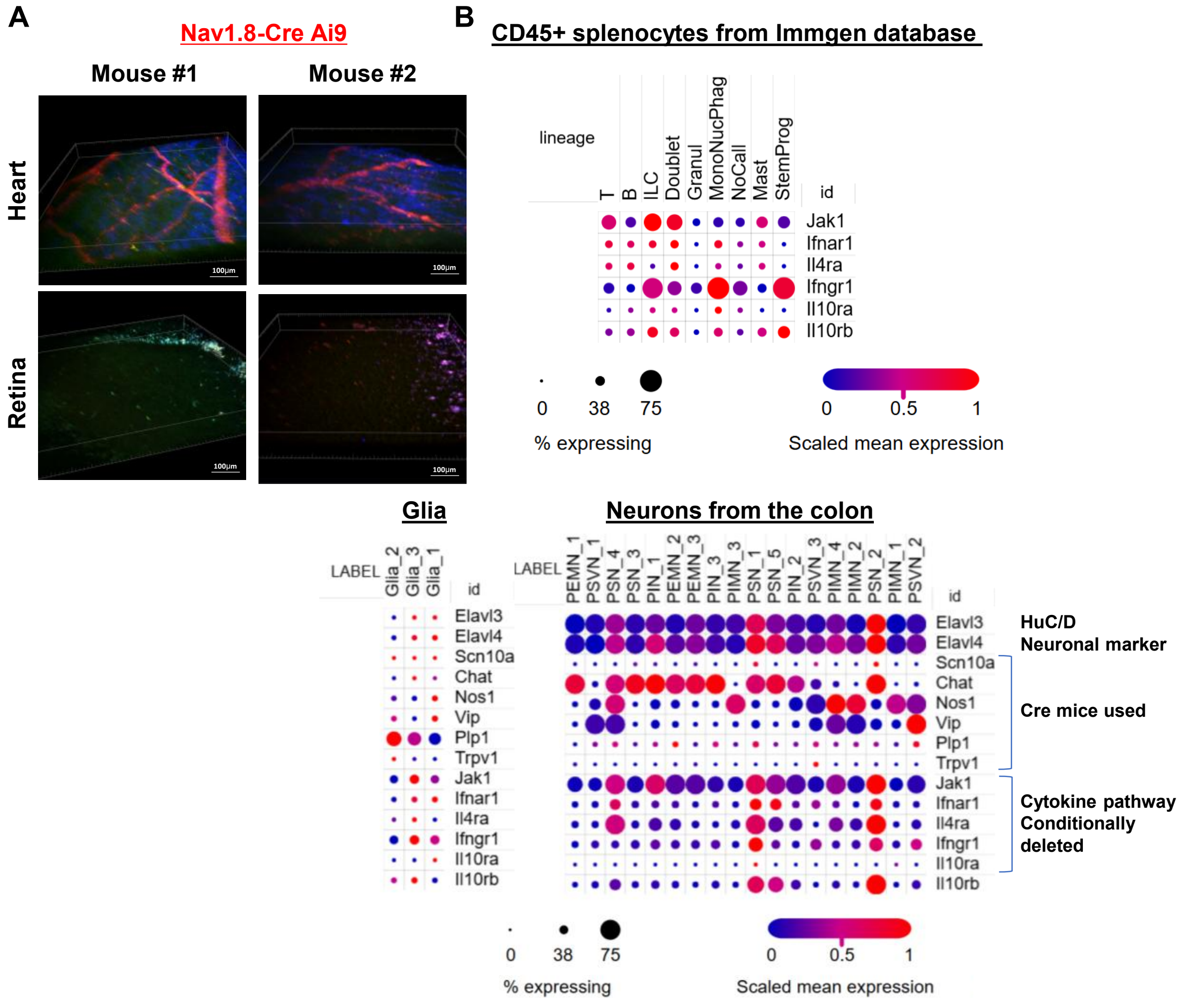

Figure S2. Expression of Nav1.8-Cre and genes relevant to this manuscript in ENS neurons, related to Figure 2

(A) TdTomato expression (red) in the heart or retina of Nav1.8-Cre Ai9 mice using two photon microscopy. The image of the retina is deeper in mouse #2. The green is from Foxp3^{GFP} in mouse #1. Scale bars represent 100μm.

(B) Using a published scRNAseq dataset (Single cell portal accession SCP1038), the expression of the indicated genes in glia (bottom left) and enteric neurons (bottom right) and are shown. This includes the neuronal marker *Elavl3/4* (HuC/D); genes related to Cre drivers used including *Scn10a* (Nav1.8), *Chat*, *Nos1*, *Vip*, *Plp1*, and *Trpv1*; and floxed genes such as *Jak1*, *Ifnar1*, *Il4ra*, *Ifngr1*, and *Il10ra*. Splenocyte

data to illustrate the low expression of *Il10ra* (top) was obtained from (Single cell portal accession SCP306).

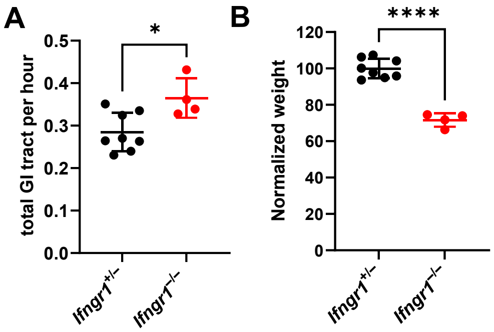

Figure S3. Impact of IFNγ on GI transit and body weight, related to Figure 3

Analysis of germline *Ifngr1*$^{-/-}$ mice compared with littermate *Ifngr1*$^{+/-}$ controls

(A) Total GI transit (*N*=8 *Ifngr1*$^{+/-}$, 4 *Ifngr1*$^{-/-}$, expt.= 2).

(B) Normalized body weight (*N*= 8 *Ifngr1*$^{+/-}$, 4 *Ifngr1*$^{-/-}$, expt.= 2).

Mice were 7- to 10-week-old at the time of analysis.

Each dot represents an individual mouse. *N* is the total number of mice.

Significance was determined by unpaired two-tailed Student's *t* test. Data are presented as mean ±SEM.

**P*<0.05, *****P*<0.0001.

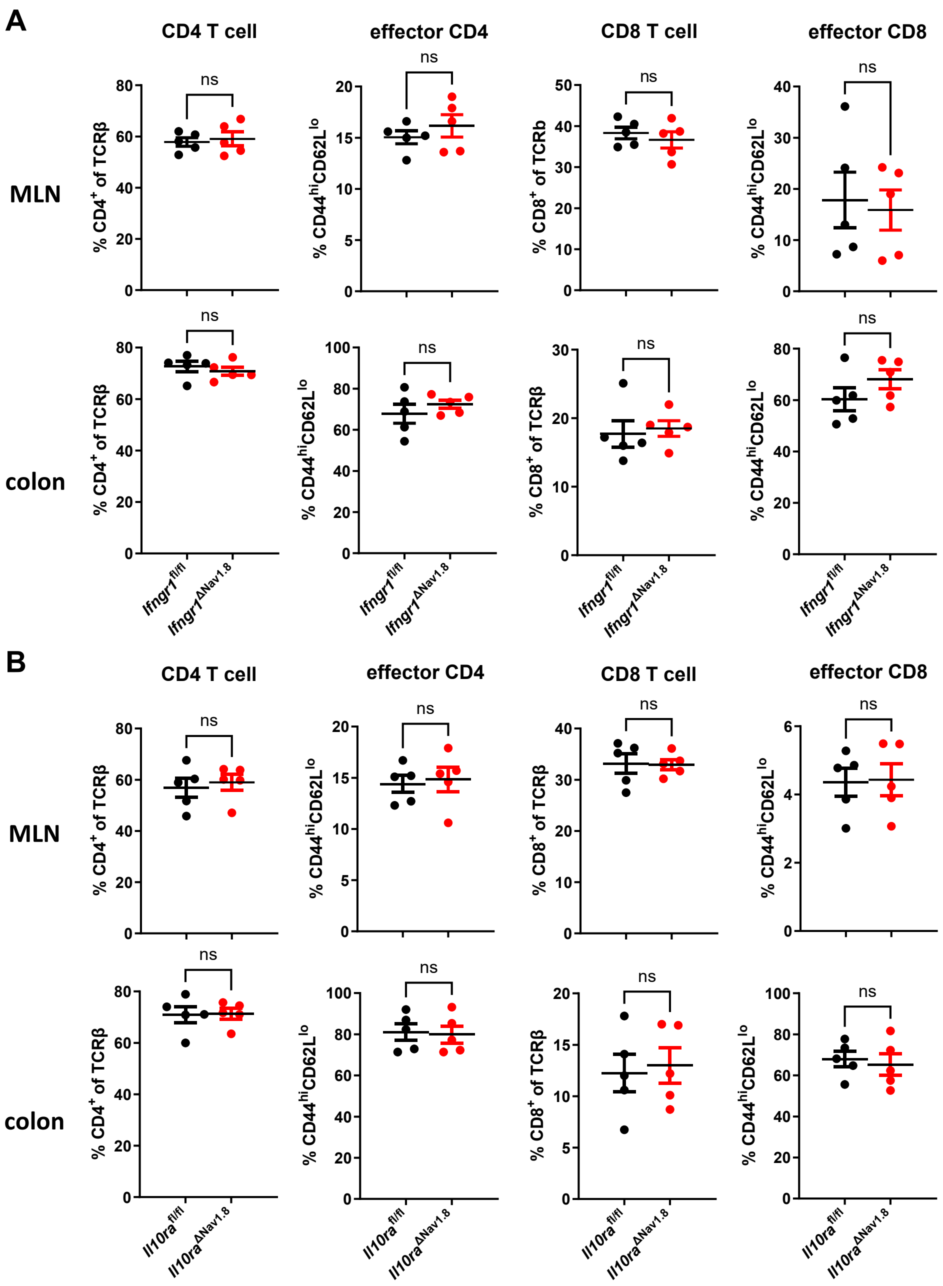

Figure S4. Nav1.8-Cre induced deficiency of IFNγ or IL-10 signaling does not alter immune cell frequencies, related to Figure 3

T cell phenotypes in the MLN (upper) or colon (lower).

(A) Nav1.8-Cre *Ifngr1*$^{fl/fl}$ (*N*= 5 *Ifngr1*$^{fl/fl}$, 5 *Ifngr1*$^{\Delta Nav1.8}$, expt.= 2) mice.

(B) Nav1.8-Cre *Il10ra*$^{fl/fl}$ (*N*= 5 *Il10ra* $^{fl/fl}$, 5 *Il10ra*$^{\Delta Nav1.8}$, expt.= 2) mice.

Mice were 7- to 10-week-old at the time of analysis.

Each dot represents an individual mouse. *N* is the total number of mice.

Significance was determined by unpaired two-tailed Student's *t* test. Data are presented as mean ±SEM. ns, not significant.

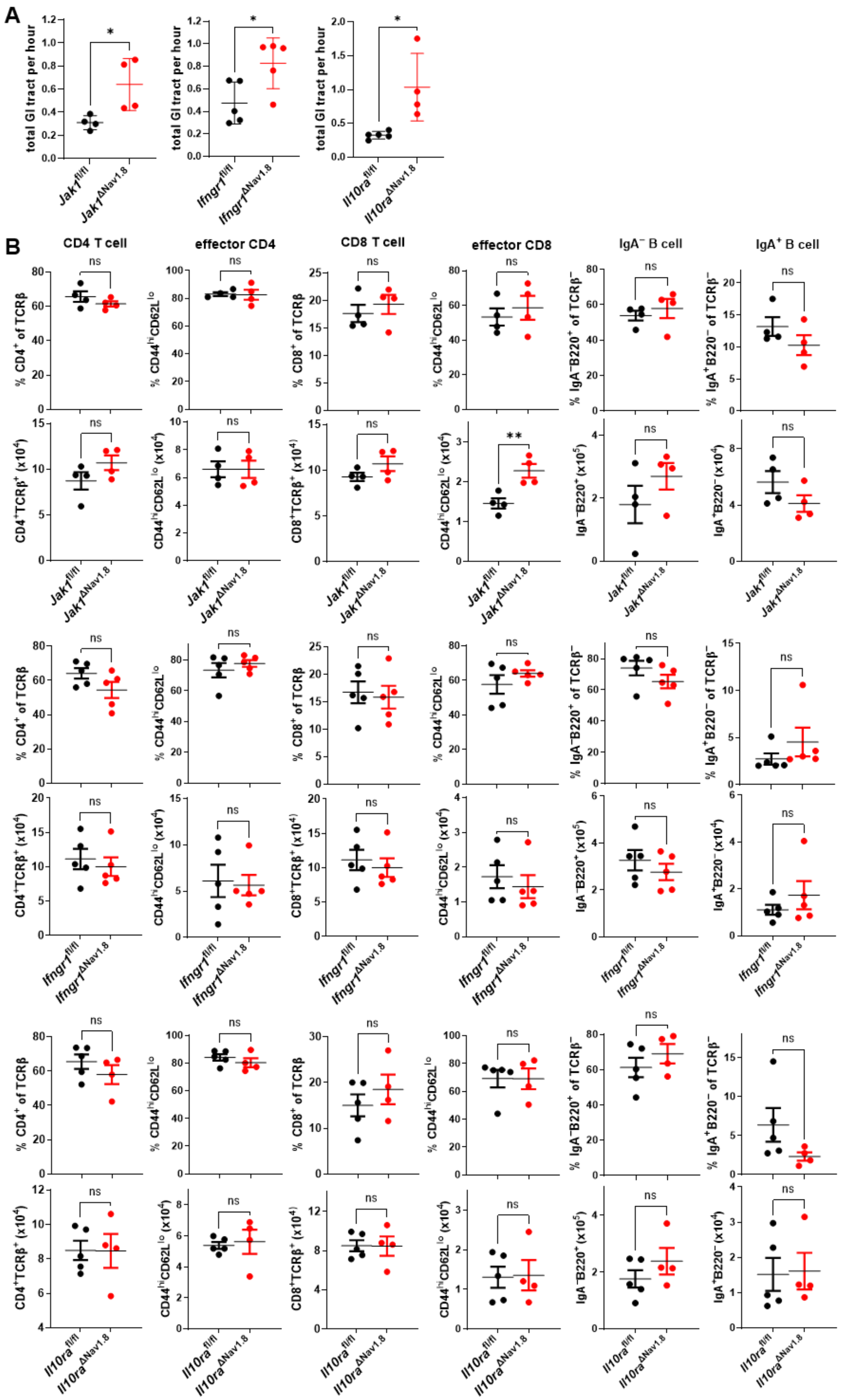

Figure S5. Additional analysis of DSS colitis, related to Figure 4

Ten-week-old Nav1.8-Cre mice bred to *Jak1*$^{fl/fl}$, *Ifngr1*$^{fl/fl}$*, or Il10ra*$^{fl/fl}$ were given 2% DSS in drinking water for 7 days.

(A) Total GI transit on day 7 after DSS (*N*= 4 *Jak1*$^{fl/fl}$, 4 *Jak1*$^{\Delta Nav1.8}$; 5 *Ifngr1* $^{fl/fl}$, 5 *Ifngr1*$^{\Delta Nav1.8}$; 5 *Il10ra* $^{fl/fl}$, 4 *Il10ra*$^{\Delta Nav1.8}$, expt.= 2).

(B) Immune cell phenotyping in the colon by flow cytometry on day 10 after DSS (*N*= 4 *Jak1*$^{fl/fl}$, 4 *Jak1*$^{\Delta Nav1.8}$; 5 *Ifngr1* $^{fl/fl}$, 5 *Ifngr1*$^{\Delta Nav1.8}$; 5 *Il10ra* $^{fl/fl}$, 4 *Il10ra*$^{\Delta Nav1.8}$, expt.= 2).

Each dot represents an individual mouse. *N* is the total number of mice.

Significance was determined by unpaired two-tailed Student's *t* test. Data are presented as mean ±SEM.

**P*<0.05, ***P*<0.01, ns, not significant.
